# Success or failure of critical steps in community case management of malaria with rapid diagnostic tests: a systematic review

**DOI:** 10.1186/1475-2875-13-229

**Published:** 2014-06-12

**Authors:** Esmée Ruizendaal, Susan Dierickx, Koen Peeters Grietens, Henk DFH Schallig, Franco Pagnoni, Petra F Mens

**Affiliations:** 1 Royal Tropical Institute/Koninklijk Instituut voor de Tropen (KIT), Amsterdam, The Netherlands; 2 Institute of Tropical Medicine/ Instituut Tropische Geneeskunde (ITG), Antwerp, Belgium; 3 Nagasaki University, School of International Health Development, Nagasaki, Japan; 4 Global Malaria Programme, 20 Avenue Appia, CH 1211 Geneva 27, Switzerland

**Keywords:** Malaria, *Plasmodium falciparum*, Community health workers, Rapid diagnostic tests, Sub-Saharan Africa

## Abstract

**Background:**

Malaria still causes high morbidity and mortality around the world, mainly in sub-Saharan Africa. Community case management of malaria (CCMm) by community health workers (CHWs) is one of the strategies to combat the disease by increasing access to malaria treatment. Currently, the World Health Organization recommends to treat only confirmed malaria cases, rather than to give presumptive treatment.

**Objectives:**

This systematic review aims to provide a comprehensive overview of the success or failure of critical steps in CCMm with rapid diagnostic tests (RDTs).

**Methods:**

The databases of Medline, Embase, the Cochrane Library, the library of the ‘Malaria in Pregnancy’ consortium, and Web of Science were used to find studies on CCMm with RDTs in SSA. Studies were selected according to inclusion and exclusion criteria, subsequently risk of bias was assessed and data extracted.

**Results:**

27 articles were included. CHWs were able to correctly perform RDTs, although specificity levels were variable. CHWs showed high adherence to test results, but in some studies a substantial group of RDT negatives received treatment. High risk of bias was found for morbidity and mortality studies, therefore, effects on morbidity and mortality could not be estimated. Uptake and acceptance by the community was high, however negative-tested patients did not always follow up referral advice. Drug or RDT stock-outs and limited information on CHW motivation are bottlenecks for sustainable implementation. RDT-based CCMm was found to be cost effective for the correct treatment of malaria in areas with low to medium malaria prevalence, but study designs were not optimal.

**Discussion:**

Trained CHWs can deliver high quality care for malaria using RDTs. However, lower RDT specificity could lead to missed diagnoses of non-malarial causes of fever. Other threats for CCMm are non-adherence to negative test results and low referral completion. Integrated CCM may solve some of these issues. Unfortunately, morbidity and mortality are not adequately investigated. More information is needed about influencing sociocultural aspects, CHW motivation and stock supply.

**Conclusion:**

CCMm is generally well executed by CHWs, but there are several barriers for its success. Integrated CCM may overcome some of these barriers.

## Background

Malaria affects over 300 million people every year, with around 90% of infections occurring in sub-Saharan Africa (SSA) [1,2]. Community case management of malaria (CCMm), formerly known as home based management of malaria, is a strategy recommended by the World Health Organization (WHO) which aims at reducing the malaria burden by improving early access to malaria directed healthcare [3]. It is based on treatment of malaria cases, mainly children, by community health workers (CHWs), within the community. Different cadres exist for CHWs, but all these individuals have in common that they are part of the community, they are not professional healthcare workers but receive a short training and often work on a voluntary basis or for small compensation (although in some countries they are included in the salaried healthcare system). While fever cases were previously treated presumptively with anti-malarials, CCMm programmes are now expected to follow the WHO recommendation to treat only malaria patients with confirmed diagnosis, usually with rapid diagnostic tests (RDTs) [4]. It is expected that this will reduce unnecessary malaria treatment and increase correct diagnosis in patients suffering from other febrile illnesses. Three published reviews describe RDT use in CCMm, however, the issues discussed in these reviews are limited [5,6] and not all of the important literature was included [6], or CHWs were not distinguished from professional healthcare providers [7]. Systematically obtained information on the success or failure of critical steps in RDT-based CCMm is lacking, but is needed in order to show its value in malaria control programmes. Furthermore, the WHO currently advises to proceed to integrated community case management (iCCM) [8], which focuses on the diagnosis and treatment of multiple diseases such as malaria, pneumonia and diarrhoea, and lessons from CCMm should be used during this transition. This systematic review aims to provide a comprehensive overview of the success or failure of critical steps in CCMm with rapid diagnostic tests (RDTs).

## Methods

### Search methodology

A systematic search was performed in the databases of Medline, Embase, the Cochrane Library and the library of the ‘Malaria in Pregnancy’ consortium (MIP consortium). Web of Science was used to search for missed relevant studies in references and citing articles. The databases were last searched on October 12, 2013. Synonyms for ‘malaria’, ‘RDT’ and ‘CHW’ were combined to find all relevant studies. For the complete search syntax see Additional file 1.

### Selection of studies

After removal of duplicates, title and abstract of articles were screened for in- and exclusion criteria by two independent readers (ER and PFM). A second screening was performed on full text articles. Discrepant results were resolved by discussion until a unanimous decision was reached. Criteria for inclusion were: original studies on RDT-based CCMm performed by CHWs defined as non-professional healthcare workers working within a community, studies on *Plasmodium falciparum* malaria, studies on one of the following critical steps: test performance by CHWs, execution of test, test interpretation, adherence to test results by CHWs, effect on morbidity and mortality, adherence to test results by patients, referral completion, social acceptance, community uptake, stock-outs, CHW incentives and motivation and cost-effectiveness. Exclusion criteria were: studies on iCMM in which the individual effect of RDT-based CCMm on the outcome cannot be identified and studies outside SSA. The focus on SSA was chosen because of the specific malaria epidemiology with a high burden of *P. falciparum* malaria and high morbidity and mortality rates [9]. Authors of relevant conference abstracts were contacted for more detailed results and information on methodology; in case of no additional information, no response, or if full study details did not meet in- or exclusion criteria, these abstracts were excluded.

### Data extraction

Data were extracted from included studies by two independent readers (ER and PFM) and additionally for social-behavioural themes by SD and KPG. Critical appraisal was done in Review Manager [10] for intervention and diagnostic studies. Evers checklist was used for studies on cost-effectiveness according to Cochrane advice [11]. For all other studies, criteria are not well defined in literature, so criteria were defined per outcome. No studies were discarded based on qualitative assessment. If possible, separate data for CCMm from studies on integrated CCM (iCCM) were extracted.

### Analysis

A forest plot was created of the RDT test characteristics by the use of Review Manager. No meta-analysis was performed due to heterogeneity of study characteristics and outcomes. The same heterogeneity accounts for adherence to test results. For the analysis of social-behavioural themes, NVivo Software for qualitative data analysis (QSR International Pty Ltd Cardigan, UK) was used.

## Results

### Search results

**Figure 1:**
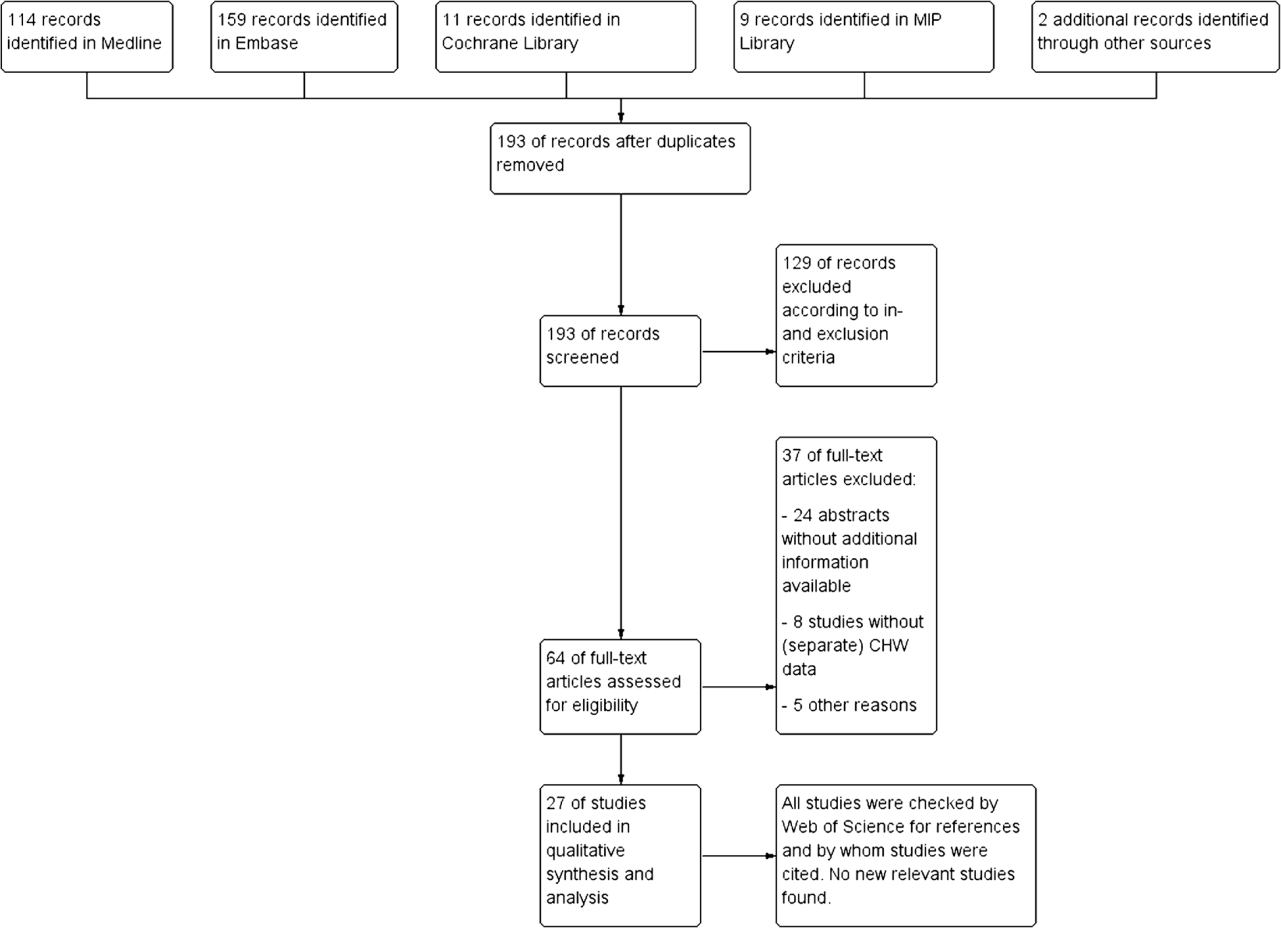
Flow chart of search strategy.

In total, 295 articles were found, of which 293 were by database searching and two via other sources. The latter articles were found after e-mail contact with authors of two abstracts [12,13]. Both referred to an article that could not be found in any of the databases [14,15]. After removal of duplications and after the screening steps on inclusion and exclusion criteria 27 articles remained for data extraction (Figure 1). Exact reasons for exclusion of full text articles can be found in Additional file 2. One article [16] provided the same outcome information as another [17] (based on the same study) and is, therefore, not separately discussed.

In Additional file 3, an overview of the included studies is presented and for each of the critical steps the number of articles and relevant articles are indicated. Most of the articles were published between 2008 and 2013.

### Risk of bias summary

**Figure 2:**
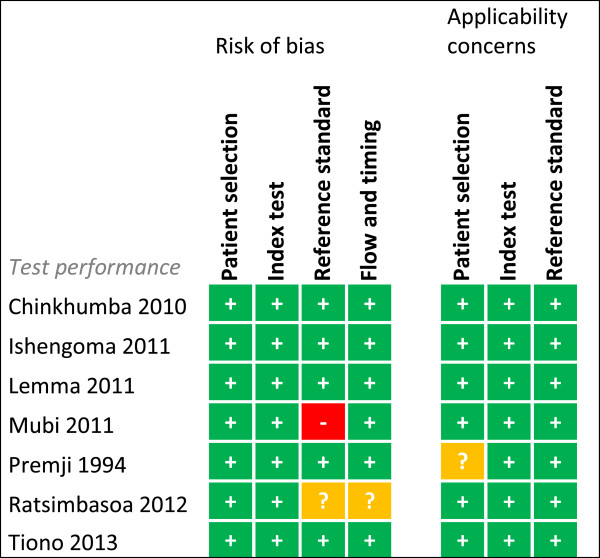
**Risk of bias summary for test performance.** + = low risk of bias, ? = unclear, - = high risk of bias.

**Figure 3:**
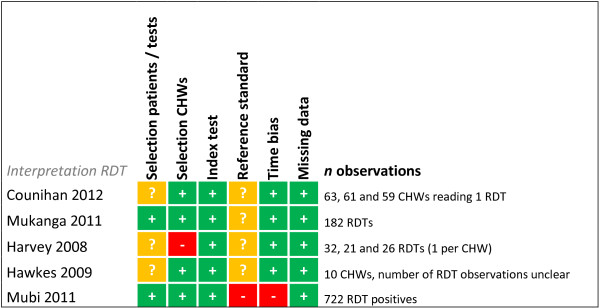
**Risk of bias summary for direct interpretation of RDT.** + = low risk of bias, ? = unclear, - = high risk of bias.

**Figure 4:**
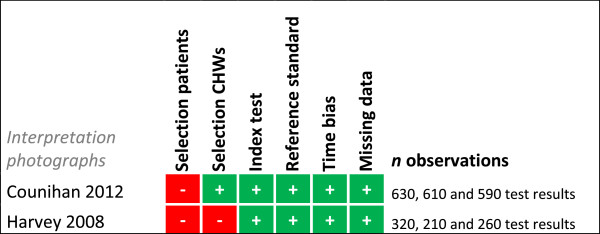
**Risk of bias summary for interpretation of photographs.** + = low risk of bias, ? = unclear, - = high risk of bias.

**Figure 5:**
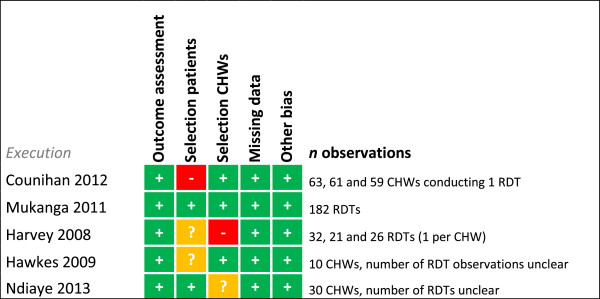
**Risk of bias summary for RDT execution.** + = low risk of bias, ? = unclear, - = high risk of bias.

**Figure 6:**
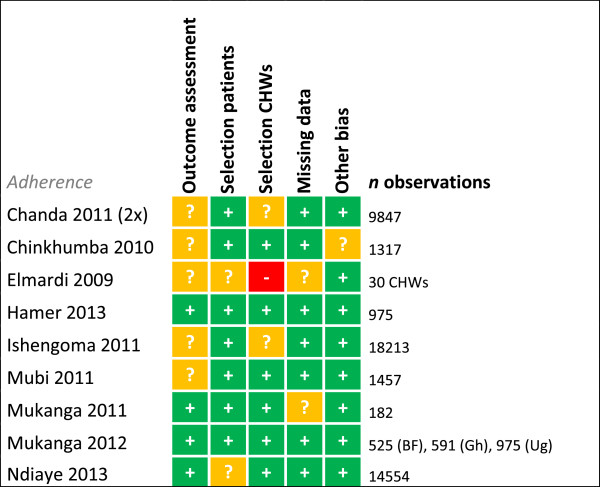
**Risk of bias summary for adherence.** + = low risk of bias, ? = unclear, - = high risk of bias.

**Figure 7:**
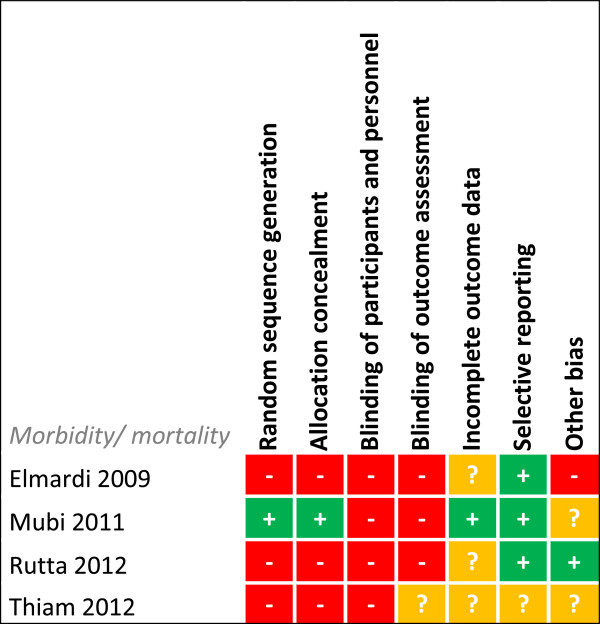
**Risk of bias summary for intervention studies on morbidity and mortality.** + = low risk of bias, ? = unclear, - = high risk of bias.

**Figure 8:**
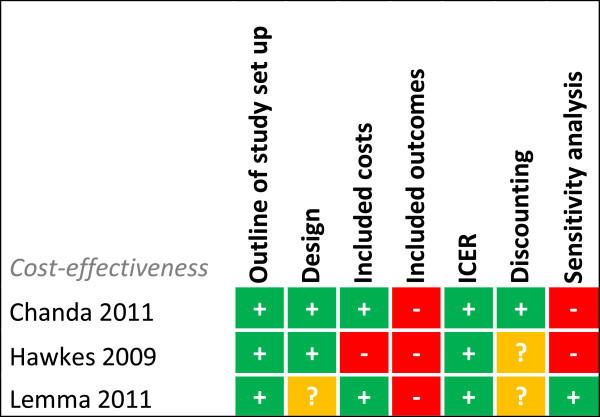
**Risk of bias summary for cost-effectiveness studies.** + = low risk of bias, ? = unclear, - = high risk of bias.

**Figure 9:**
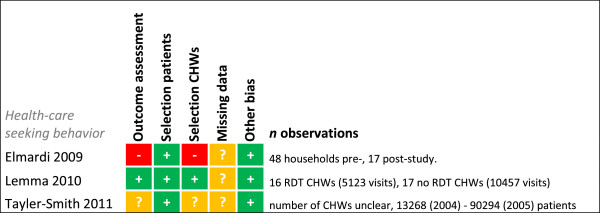
**Risk of bias summary for healthcare-seeking behaviour.** + = low risk of bias, ? = unclear, - = high risk of bias.

Risk of bias was assessed for each study for every outcome (Figures 2, 3, 4, 5, 6, 7, 8 and 9). Nearly all studies on RDT performance showed low risks of bias. The same accounted for RDT execution, although Counihan *et al.*[18] sometimes used non-febrile volunteers instead of patients for RDT observations and Harvey *et al.*[19] showed baseline differences in the groups of CHWs. Adherence to test results showed little bias, although it was often unclear how studies collected data. One study on adherence showed a possible patient selection bias because a large group of malaria-suspected patients was not tested at all [20]. More variable quality was found in studies on interpretation, healthcare-seeking behaviour, stock-outs and cost-effectiveness. In all three cost-effectiveness studies, debatable assumptions were made: sensitivity and specificity used for calculations were derived from studies on professional healthcare workers instead of CHWs [21-23]. Furthermore, in two cost-effectiveness analyses adherence to test results was assumed to be 100% [21,22]. High risk of bias was seen for all studies concerning morbidity and mortality; none met the preferred intervention design of a double-blind, randomized, controlled trial (RCT). At the same time, blinding patients and CHWs is impossible for this type of intervention. It is therefore unfortunate that the one study that described a non-blinded cluster RCT used a subjective outcome measure (patients’ reporting of clinical recovery), potentially introducing bias [24]. The other studies were either not randomized [25], or were single-armed, pre- and post-intervention studies [26,27]. It should be noted that studies that showed high risk of bias for a certain outcome did not necessarily show high risk of bias for their main objective.

### RDT performance when used by CHWs

**Figure 10:**
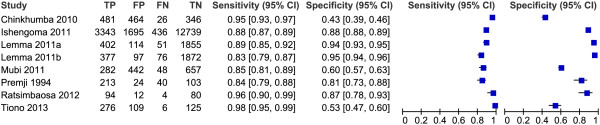
**Forest plot of RDT performance when performed by CHWs (no subgroup analyses).** Lemma 2011a = Paracheck Pf, Lemma 2011b = Parascreen pan/p.

Sensitivity and specificity of RDTs when performed by CHWs was assessed in seven studies (Figure 10 and Table 1). Studies differed in participants, malaria transmission and RDT type used. Sensitivities ranged between 83.2 and 97.9% if RDTs were compared with microscopy as reference standard. Ishengoma *et al.* found that the sensitivity was significantly higher for cases < five years of age and for fever cases [28]. Sensitivity decreased with decreasing malaria transmission over the years in this study [28], but this was not confirmed in Tiono *et al*. [14]. Ratsimbasoa *et al.* additionally calculated RDT sensitivity with PCR as reference standard (RS) and found a sensitivity of 61.8% [29].

**Table 1:** RDT test performance by CHWs

**Study**	**Target population**	**RDT type**	**Reference standard (RS)**	RS positive* (%)	**Sensitivity (%)**	**Specificity (%)**
**Ratsimbasoa**[29]	>2 months (history of) fever	CareStart	PCR	56.7%	61.8%	95%
**Ratsimbasoa**[29]	>2 months (history of) fever	CareStart	Microscopy of thin and thick BS	37.2%	95.9%	87%
**Chinkhumba**[30]	>5 years (history of) fever	Bioline SD, First Response malaria, Paracheck PF	Microscopy of thick BS (expert)	38.5%	95%	43%
**Chinkhumba**[30]**	>5 years (history of) fever	Bioline SD	Microscopy of thick BS (expert).	41%	97%	39%
**Chinkhumba**[30]**	>5 years (history of) fever	First response malaria	Microscopy of thick BS (expert).	40%	92%	42%
**Ishengoma**[28]	All ages, care seeking	Paracheck Pf, ParaHIT	Microscopy of thick and thin BS.	20.8%	88.6%	88.2%
**Ishengoma**[28]	< 5 years	Paracheck Pf, ParaHIT	Microscopy of thick and thin BS	19.7%	90.1%	93.6%
**Ishengoma**[28]	≥5 years	Paracheck Pf, ParaHIT	Microscopy of thick and thin BS	21%	88.3%	86.5%
**Ishengoma**[28]	No fever patients	Paracheck Pf, ParaHIT	Microscopy of thick and thin BS	14.5%	84.7%	90.1%
**Ishengoma**[28]	Fever patients	Paracheck Pf, ParaHIT	Microscopy of thick and thin BS	33.9%	92.2%	82.9%
**Lemma**[22]**	>3 months suspected of malaria	Paracheck Pf	Microscopy of thick BS.	18.7%	88.7%	94.2%
**Lemma**[22]**	>3 months suspected of malaria	Parascreen	Microscopy of thick BS.	18.7%	83.2%	95.1%
**Mubi**[24]	>3 months (history of) fever.	Paracheck Pf	Microscopy of thick BS.	22.6%	85.3%	59.8%
**Premji**[31]	Children <42 months	Parasight TM-F test	Microscopy of thin and thick BS.	66.6%	84%	81%
**Tiono**[14]	Children 6–59 months with (history of) fever.	FirstSign Malaria Pf	Microscopy of thin and thick BS.	54.8%	97.9%	53.4%
**Tiono**[14]	Children 6–59 months with (history of) fever. (High transmission)	FirstSign Malaria Pf	Microscopy of thin and thick BS.	76.1%	98%	25.4%
**Tiono**[14]	Children 6–59 months with (history of) fever. (Low transmission)	FirstSign Malaria Pf	Microscopy of thin and thick BS.	31.8%	97.6%	63.7%

*P. falciparum, **Two types of RDTs separately tested.

The specificity of RDTs in the hands of CHWs was found to be more variable than the sensitivity, ranging from 39% in the study of Chinkhumba *et al*. [30] to 95.1% in the study of Lemma *et al*. [22]. In Chinkhumba *et al*. [30] patients who self-treated with anti-malarials (2-8% in previous two weeks) were not excluded and this was associated with a lower specificity. Mubi *et al*.[24] also showed low specificity, but it was mentioned that the microscopy slides were of poor quality, possibly impairing the detection of parasites in actual true positive samples. Slide quality was not mentioned in Tiono *et al*. [14], who found a low specificity, especially in the high transmission season (25.4%). The conditions for RDT storage were appropriate and the authors mainly related the results to persistent circulating antigens [14]. In Ishengoma *et al*. a higher specificity for children < five years of age was shown compared with older patients [28].

### RDT interpretation

RDT interpretation was assessed by either direct assessment of RDTs (five studies) or by photographic assessment (two studies) (see Table 2). Correct interpretation was high for the direct assessment of RDTs; 96 to 100% of tests and 95.1 to 100% of CHWs, provided that CHWs were trained properly [18,19,21,32]. In photographic assessments, which have the inherent risk of selection bias as the number of ambiguous tests is relatively high, well-trained CHWs also scored high numbers of correctly interpreted tests, although more mistakes were made in reading faint positive and invalid tests [18,19]. The importance of training was shown for direct interpretation of RDTs as well as for photographic assessment in Harvey *et al*. [19]. In both assessments correct interpretation was lowest for CHWs who used only manufacturer’s instructions, better for CHWs who used a job aid and the best for CHWs who used a job aid and received three hours’ training. Unfortunately, a risk of selection bias was present because CHWs were not randomly selected and completion of secondary education was 6, 19 and 35%, respectively. Some indications were also found for the beneficial effect of repetitive execution of RDTs. In Harvey *et al*. improvement was seen with each successive RDT, although these data were not presented [19]. Improvement over time was also presented by Counihan *et al*. [18]; the percentage of CHWs correctly interpreting RDT results from patients or volunteers at three, six and 12 months after training was 95.1, 98.3 and 98.3%, respectively. In contrast, this was not the case with the ten-item photographic assessment in which four CHWs consistently showed poor interpretation.

**Table 2:** Interpretation and execution of RDTs by CHWs

**Study**	**CHW training**	**Outcome interpretation**	**Outcome execution**
**Counihan**[18]	Half-day training. At 3 months CHWs received a poster-sized job aid and a photographic guide on RDT interpretation.	(I) RDT test results correctly read by 95.1, 98.3 and 98.3% of the CHWs at 3, 6 and 12 months after training respectively.	19-item checklist, interpretation included, 8 items were considered critical.
		(II) Correct interpretation of positive RDT results was 96.5% at 3 months, 98.3% at 6 months and 90.5% at 12 months.	Median correctly performed critical steps were 87.5%, 100% and 100% at 3, 6 and 12 months respectively.
		(II) Correct interpretation of negative RDT results was 94.3% at 3 months, 97.9% at 6 months and 94.7% at 12 months.	40.3, 61.7 and 79.7% of CHWs correctly performed critical RDT steps at 3, 6 and 12 months respectively.
		(II) Faint positive lines were correctly interpreted by 89.7% at 3 months, 96.7% at 6 months and declined to 76.7% at 12 months.	
**Mukanga**[32]	8-day training by experienced trainers. Job aid provided.	100% of the RDTs were correctly interpreted shortly after training (<2 weeks).	96.3% of RDTs were correctly performed shortly after training (<2 weeks) in a 14-item checklist, interpretation excluded.
**Harvey**[19]	Group 1: only use of manufacturers’ instructions. Group 2: only use of job aid. Group 3: 3- hour training on RDTs + job aid.	(I) 72, 86 and 96% of CHWs correctly interpreted RDT results for group 1, 2 and 3 respectively.	57% of steps, 80% of steps and 90% of steps were correctly performed by group 1, 2 and 3 respectively at the same day of receiving instructions, job aid or training in a 16-item checklist, interpretation included.
		(II) 54, 82 and 93% of tests were correctly interpreted for group 1, 2 and 3 respectively.	
**Hawkes**[21]	One day training. Pictorial job aid was provided.	100% of CHWs correctly interpreted the RDT directly after training.	Median score on a WHO 16-item assessment of RDT performance was 100% (range of 94-100%) directly after training.
**Ndiaye**[20]	CHWs: one month theoretical training, one month practical training at health post. CMDs: 3-day theoretical training, 15 days practical training at health post.	-	*% CHWs and CMDs correctly performing the step, observed over two years.*
			(1) Surface clean and flat - 87%
			(2) Test opened just before use - 100%
			(3) Document patient name and date - 83%
			(4) Use of gloves - 0%
			(5) 5 μL finger prick blood specimen - 93%
			(6) 4 drops of solution buffer in right well - 93%
			(7) test rest on level surface - 97%
			(8) waited maximum 15 minutes - 93%
**Mubi**[24]	One week training.	99.7% of positive tests were correctly interpreted throughout the 5-month study period.	-

(I) Based on assessment of RDTs.

(II) Based on photographic assessment.

### Execution of RDTs

Execution of RDTs was investigated in five studies that used non-uniform outcome variables (see Table 2). The way CHWs executed RDTs was judged on several items, but differences in number and definition of items further impaired comparison [18-21,32]. Nevertheless CHWs were found to correctly conduct RDTs if properly trained; 90 to 100% of steps were successfully executed in two studies that used the same WHO checklist, Hawkes *et al*. [21] and Harvey *et al*. [19]. In contrast, untrained CHWs that used only manufacturer’s instructions or a job aid were found to respectively conduct only 57 and 80% of steps correctly in Harvey *et al.*[19]. The assessment of each individual executed step in this study showed that problems were found in dispensing buffer drops, waiting correct amount of time, pricking side of finger, blood collection and recording result in register. Counihan *et al*. [18], who assessed only the execution of eight RDT steps (see Additional file 4) that were considered critical for diagnosis or safety, showed improvement over time: 40.3, 61.7 and 79.7% of CHWs correctly performed critical steps at three, six and 12 months, respectively. Problematic steps were writing patient’s name on cassette, recording results in register, usage of the blood collection loop, reading the test result in the right time and disposing non-sharps in non-sharps container [18-20]. Why these steps were problematic was not studied. No specific studies were done that looked at safety issues while performing RDTs but some included particular findings. Ndiaye *et al*. [20] observed that 0% of CHWs used gloves, but this was partly due to stock problems. In contrast, high levels of glove use were reported in Counihan *et al*. [18] (100% of CHWs after 12 months) and Harvey *et al*. [19] (96% in the group of trained CHWs). In Counihan *et al*. [18] an observer had to intervene once because a CHW was about to re-use a lancet on a new patient.

### Adherence to test results and referral guidelines by CHWs

In general almost all patients (>90.0%) with positive RDTs were provided with anti-malarial drugs by CHWs (Table 3). The percentage of patients with negative RDTs who, contradictory to the guidelines, still received anti-malarial treatment was more variable, ranging from 0.2 to 58%. Six studies showed levels <10%, including all iCCM studies, while two studies showed outliers of 20.3% [20] and 58% [30] of negative-tested patients that were treated. One other study presented the percentage of CHWs adhering to test results and showed that 30% of them treated negative-tested patients based on clinical judgment rather than RDT result; this was not related to previous experience in malaria management or educational background [27]. Ndiaye *et al*. [20] showed that the adherence was related to the type of care providers in their study. Community medicine distributors (CMDs) were only trained on RDT-based malaria management, while CHWs were attributed more health intervention tasks. CHWs gave artemisinin-combination therapy (ACT) to 24.8% of RDT negatives, while CMDs gave ACT to 10.4% of RDT negatives. Besides treating a high number of negative patients, CHWs also treated 22.3% of patients with ACT who were not tested at all, while for CMDs this was only 0.8%. Furthermore, CHWs and CMDs did not comply with the referral policy; referral rates from patient groups that should have been referred ranged from 18.2 to 47.1% with the lowest referral rates found for babies < two months of age and patients with severe symptoms. The only other study reporting on referral by CHWs showed better results, in the first year, 79.5% of RDT negatives were referred according to protocol and in the second year this had increased to 97.4% [25]. No explanations were given in the studies on reasons for non-adherence regarding treatment of negatives and referral.

**Table 3:** Adherence to test results by CHWs

**Study**	**Target population**	**Treatment**	**Alternative**	Adherence overall*	**Positives treated**	**Negatives treated**
***CCMm studies***						
**Chanda**[23,33]	All ages, care seeking.	AL, SP <5 kg	Complicated malaria and non-malaria febrile cases were referred to HF.	99.9%	99.3%	0.2%
**Chinkhumba**[30]	>5 years, (history of) fever	NS	Referral not mentioned.	86.9%	98%	58%
**Elmardi**[27]	NS	AS/SP	Complicated malaria and non-malaria febrile cases were referred to HF.	70%**	NS	NS
**Ishengoma**[28]	≥5 years with (history of) fever	AL	Referral not specified.	95.8%	98.9%	5.4%
**Mubi**[24]	>3 months, (history of) fever. Exclusion: severe disease	AL	Referral not specified.	96.8%	99.7%	6.1%
**Ndiaye**[20]***	Patients of all ages, care seeking.	NS	CHW: referral of patients <2 months, RDT negatives, severe symptoms, suspected drug adverse events. CMD: referral of all cases excluding uncomplicated malaria cases.	88.6%	92.0%	20.3%
**Ndiaye**[20]***	Patients of all ages, care seeking.	NS	CHW: Referral of patients <2 months, RDT negatives, severe symptoms, suspected drug adverse events.	85.6%	90.1%	24.8%
**Ndiaye**[20]***	Patients of all ages, care seeking.	NS	CMD: Referral of all cases excluding uncomplicated malaria cases.	93.9%	95.3%	10.4%
***iCCM studies***						
**Hamer**[17] & **Yeboah-Antwi**[16]	Children 6 months-5 years, fever.	AL	Children with danger signs were referred to HF.	99.3%	98.5%	0.4%
**Mukanga**[32]	Children <5 (history of) fever no danger signs.	AL	CHWs also diagnosed and treated pneumonia. No referral mentioned.	97.8%	98.6%	4.8%
**Mukanga**[34]****	BF: 6–59 months, (history of) fever	AL	Referral for severe disease and for non-responders at day 3 after CHW visit.	99.0%	100%	4.8%
**Mukanga**[34]****	Gh: 6–59 months, (history of) fever	AA	Referral for severe disease and for non-responders at day 3 after CHW visit.	99.5%	100%	3.3%
**Mukanga**[34]****	Ug: 4–59 months (history of) fever.	AL	Referral for severe disease and for non-responders at day 3 after CHW visit.	99.0%	99.9%	7.6%

* = correct treatment, ** = percentage of CHWs that relied on RDT results, *** = adherence percentages calculated from study data, **** = CHWs attended review meetings with study team each month discussing non-adherence to diagnostic and treatment algorithm with CHWs, HF = health facility, NS = not specified, AL = artemether – lumefantrine, SP = sulphadoxine-pyrimethamine, AS = artesunate, ACT = artemisinin-based combination therapy (not further specified), BF = Burkina Faso, Gh = Ghana, Ug = Uganda.

### Morbidity and mortality

Four trials assessed outcomes related to morbidity, mortality or both (Table 4), but all showed low quality evidence (Figure 7) impairing firm conclusions. One study reported lower slide positivity rates during the RDT-based CCMm intervention period in comparison with presumptive CCMm in the pre-intervention period. However, besides changing to RDT-based diagnosis, the type of anti-malarial drug used changed from sulphadoxine-pyrimethamine to an ACT in this study [26]. Two other studies showed beneficial effects of RDT-based CCMm but only compared it to areas with no CCMm [25,27]. In contrast, the perception of patients on morbidity did not improve with RDT-based CMMm. In a randomized, cross-over trial on RDT-based CCMm *versus* presumptive CCMm, a significant increased perception of disease recovery was found in the presumptively treated patients at day 7 compared with the intervention arm (97.3 *versus* 93.3%, *p* = 0.000). More patients were treated with anti-malarials in the control group; however, in both the intervention and control group the perceived unrecovered patients harboured only few malaria positives (repeated testing at day 7), thus suggesting a possible bias in the subjective study outcome [24].

**Table 4:** Morbidity and mortality outcomes of RDT based CCMm strategies

**Study**	**Design**	**Intervention**	**Control**	**Outcome**
**Mubi**[24]	RCT	RDT-based CCMm	Presumptive CCMm	Increased perception of recovery in control group (97.3%) *versus* intervention group (93.3%) at day 7. *P* = 0.000
				Two malaria related deaths, one in each arm.
**Thiam**[25]	NRCT	RDT-based CCMm	No CCMm	Malaria related hospitalizations decreased by 43.1% in intervention areas and 40.9% in control areas. Malaria attributed deaths decreased by 62.5% in intervention areas (significant decrease) and 23.4% in control areas (no significant decrease).
**Rutta**[26]	Pre-post study	RDT-based CCMm (with AL)	Comparison with pre-intervention period (presumptive CCMm with SP)	A drop of >72.0% in malaria slide positivity rate to a persistent low level of <10% was observed in the study period.
**Elmardi**[27]	Pre-post study	RDT-based CCMm (with AS/SP).	Comparison with pre-intervention period (no CCMm, health centres treated with AS/SP)	24% fever cases in last two weeks pre-intervention and 8.5% fever cases post intervention (p = 0.000).
				61 deaths (all <5 years) in the last season pre-implementation of intervention versus 1 death (>5 years) in the season post-implementation (p = 0.000).

### Community acceptance

Studies by Mukanga *et al*. [35] in Uganda and Nsagha *et al*. [36] in Cameroon assessed the opinion of community members before introducing an RDT-based CCMm intervention. In Mukanga *et al*. [35] presumptive CCMm was already implemented and a positive attitude towards the CHWs was present, due to their voluntary services, accessibility and the effectiveness of provided drugs. The change to CHWs using RDTs was therefore well received. In Nsagha *et al*. [36] all participants would welcome RDTs, but in this urban setting, where other healthcare providers are available, CHWs were not always considered to be the appropriate persons to carry out RDTs. Participants in both studies stressed that proper training of CHWs on RDT use was considered essential.

A few years later Mukanga *et al*. [37] asked community members in Uganda for their opinion after introduction of RDT-based CCMm; 79.4% thought CHWs’ service was better after introduction of RDTs and 88.7% thought CHWs should continue to use RDTs. Support for a high acceptance was also found in Senegal, where community members mainly praised the increased access to malaria care [38]. Furthermore, in several studies participants welcomed RDTs since it made correct diagnosis possible at the village level, which saved money on transport [27,36,38].

Acceptability of diagnosis and treatment by CHWs was related to the outcome of the RDT. Only 5% of CHWs had problems persuading RDT-positive patients of a malaria diagnosis in Sudan [27]. This is supported by Mubi *et al*. [24], in which 97.4% of patients complied to prescribed treatment after a positive RDT. However, persuading patients that they did not have malaria was problematic for 20% of CHWs in case of negative test results [27], an issue that was already predicted by Mukanga *et al*. [35].

Adherence to referral advice by patients was reported in only two studies [33,39]. Chanda *et al*. [33] used a system in which CHWs were notified by health centre staff if a referred patient visited the health centre. For 40 to 42% of the referrals feedback was received. In contrast, Thomson *et al*. [39] found a very low referral completion of 1.5%. They surveyed children three to 59 months and pregnant women in their second and third trimester in Sierra Leone. A large variation in referral completion was found within the different communities (0 to 18.8%). Furthermore, if stratified for RDT results, referral completion was 88.2% for RDT positives (usually referred for signs of severe malaria) in contrast to 0.9% for RDT negatives. Barriers for adherence mentioned in focus group discussions were bad roads [38], difficulties in transport [38], distance to health centre [33,38] and lack of staff at the health centre which may result in long waiting hours [33].

### Uptake of RDT based CCMm by members of the community

Three studies reported on the uptake by the community of RDT-based CCMm in comparison to baseline levels (pre-intervention period), presumptive CCMm or health centre-based care (Table 5). Two studies showed an increased use of RDT-based CCMm services over the study period [15,27]. Additionally, one of the studies compared it to health centre-based care and showed a constant number of visits in these clinics. It was concluded that the overall access to malaria healthcare had thus increased by the intervention [15]. These positive findings are not always supported, as in a study of Lemma *et al*. [40], the number of people visiting the randomly selected CHWs who performed RDT-based CCMm was half of those visiting CHWs who used presumptive diagnosis. The reasons for this reduction were not further investigated. Obstacles for visiting the CHW mentioned by the community in three other studies were the unavailability of the CHW [16,37], dislike of CHW services [37], distrust of the skills of the CHWs [37], lack of drugs [37], fear of HIV/AIDs infection [35], a disease that is perceived to be too severe for CHW to handle [16], and the relative distance to the health centre *versus* to the CHW [16,37].

**Table 5:** Healthcare-seeking behaviour

**Study**	**Intervention**	**Control**	**Outcome**
**Elmardi**[27]	RDT-based CCMm	Comparison with pre-intervention period (no CCMm)	Pre-intervention 25% of mothers of sick children <5 years would seek care within the village, after the study 64.7% would seek care within the village (p value).
**Lemma**[40]	RDT-based CCMm	Presumptive CCMm	Only half the number of patients (5,123 patients) visited CHWs who performed RDT-based CCMm compared with presumptive CCMm (10,475 patients).
**Tayler-Smith**[15]	RDT-based CCMm free of charge	Health centre care, little payment was required for ACT.	In two years there was an increase in number of episodes of treated malaria per child per year from 0.4 to 1.2 for CHWs, whereas it remained stable at 0.2 for health centres.

### Stock-outs

In the pre-intervention focus group discussions in the study of Mukanga *et al*. [35], problems in lack of transport for replenishment of supplies were foreseen. Four studies mentioned stock-out problems [18,20,33,38]. The only other study that mentioned supply issues did not experience any stock-out problems [27]. In all studies the CHW was instructed to replenish stocks at an affiliated health centre. Blanas *et al*. [38] found in 2009 that 74% of villages did not have RDTs or the RDTs were expired. In 68% of villages no ACT was available. However, Ndiaye *et al*. [20] found in the same study area in 2010 and 2011, that 90.2% of CHWs had RDTs in stock and 88% had sufficient ACT in stock. It seems that experience over time helped stock management, although in the latter study only 11.8% of all stock management forms were completed. Stock-out problems were also found in the studies of Chanda *et al*. [33] (stock-outs of RDTs for about two weeks) and Counihan *et al*. [18] (stock-outs of drugs and RDTs). One of the biggest problems for CHWs in the latter study was the refusal of health centres to resupply the CHWs. Health centres were either not informed about the agreement on stock supply or they experienced stock-outs themselves [18].

### Motivation and remuneration of CHWs

Ndiaye *et al*. [20] showed that there was high seasonal variation in CHW participation in Senegal. This could be due to the difference in malaria burden but the authors also suspect that the little remuneration and the nearby gold mining activities had their impact on CHW participation. CHWs are mostly considered volunteers, but incentives such as small fees for their consultation [27], compensation in material form or services [16,17,23,29], or structured payments [17,24,26,30] were mentioned in most studies. In Elmardi *et al*. [27] only 35% of CHWs were satisfied with the financial outcome of their services which was 0.5 US$ per consultation. However, when these CHWs were asked about the most motivating aspect of their work, it was not the financial compensation but community respect and spiritual outcome. This was supported by Hamer *et al*. [17] in which only four of 18 CHWs received some kind of payment, but almost all were satisfied or highly satisfied with their CHW job (37 and 61%, respectively). There was no information on the impact of incentives and motivation on the attrition of CHWs.

### Cost-effectiveness

Two studies on cost-effectiveness compared RDT-based CCMm to presumptive CCMm [21,22] and a third compared it with health centre- based malaria care [23] (Table 6). All three studies did not have sufficient quality for cost-effectiveness analyses as described in risk of bias summary and all used a short-term outcome for health benefit, that is cost per correctly treated case [22,23], or related cost per case saved from unnecessary treatment [21]. Compared to health centre-based care, costs for RDT-based CCMm were lower per correctly treated case, but the additional cost per change in case appropriately diagnosed and treated was 4.18 US$ for RDT-based CCMm in Zambia [23]. RDT-based CCMm was also found to cost less compared with presumptive CCMm for areas with low to medium malaria transmission [22], but not in high transmission areas as was shown in the Democratic Republic of Congo [21]. Furthermore, study region could be of importance as shown by Lemma *et al*. [22] in Ethiopia. The study was situated in an area where *Plasmodium vivax* is also prevalent and consequently the RDT that differentiated between types of malaria species showed the lowest costs per appropriately treated case. However, for total costs, only differentiating between *P. falciparum* malaria and all other fever cases was the cheapest option.

**Table 6:** Cost-effectiveness of RDT based CCMm strategies

**Study**	**Intervention**	**Control**	**Malaria prevalence**	**Outcome**
**Hawkes**[21]	RDT-based CCMm for ≥5-14 years, presumptive <5 years old.	Presumptive treatment up to 14 years old.	88% by microscopy, for calculations prevalence of 80% was considered.	8.79 US$ for each case saved from unnecessary treatment (total health budget per person per year is 15$). Total costs three times as high for RDT based CCMm.
**Lemma**[22]	RDT-based CCMm for *P. falciparum* with AL, other febrile cases treated with CQ.	Two comparisons. 1. RDT-based CCMm for *P. falciparum* (AL) and *P. vivax* (CQ) and referral of all others.	Slide positivity rate 27.29%, of which 70% *P. falciparum.*	Intervention: 4.66 US$ per correctly treated case.
				Control 1. 1.69 US$ per correctly treated case.
				Control 2. 11.08 US$ per correctly treated case.
		2. Presumptive treatment with AL for all fever patients.		
				Total costs were lowest for intervention strategy.
**Chanda**[23]	RDT-based CCMm with AL for all age groups (free of charge)	Health centre-based care (free of charge)	Prevalence 24% in RDT-based CCMm and 26% in health centres, either by RDT or microscopy.	Cost per case appropriately diagnosed and treated 4.22 US$ in RDT based CCMm (mainly because of higher adherence) and 6.61 US$ in health centers. Additional cost per change in case appropriately diagnosed and treated was 4.18 US$.

## Discussion

This review showed that CHWs are able to provide qualitative health care for malaria if properly trained. This is substantiated by other reviews for shared outcomes [5-7]. Nevertheless some barriers are present for the success of the intervention. The success and failure of each of the steps in CCMm is discussed below.

The high quality of care is reflected in the good sensitivity and specificity of RDTs used by CHWs compared with microscopy. A high sensitivity is needed because, as the new first line of health care, RDT-based CCMm cannot be inferior in detecting malaria cases to the existing practices in health centres in which microscopy is often used, especially because it seems that RDT negatives, including false negatives, may not always reach a health centre for additional care [33,39]. When the RDTs performed by CHWs were compared to PCR the sensitivity lowered but many of these cases were also missed by microscopy [29]. Furthermore, the clinical relevance of these low parasitaemia cases is being debated, because they are considered to be ubiquitous and usually asymptomatic, although it might cause anaemia [41]. Recent indications of a decreased malaria transmission in regions in SSA could however lead to lower levels of immunity in the population and might subsequently increase disease susceptibility, also for subjects with low parasitaemia levels [42]. RDT performance in case of low parasitaemia levels will therefore become increasingly important, especially in elimination settings.

Specificity levels were more variable, which is also seen in RDT use by professional health-care workers [43]. This is a potential threat to the success of CCMm as it could lead to overtreatment and missed diagnoses of other febrile illnesses. This could be a reason to promote the implementation of iCCM, as other serious causes of fever, such as pneumonia, will be detected with this intervention. Factors influencing specificity could be microscopy slide quality [24] and persistent circulating HRP2 antigens (the diagnostic target of *P. falciparum* RDTs) after clearance of the malaria infection [44]. The latter might explain a lower specificity when a substantial part of individuals received previous anti-malarial treatment [30] and the lower specificity for individuals with a higher immunity against malaria, such as children > five years of age in Ishengoma *et al*. [28] and in Chinkhumba *et al*. [30]. Another possibility for a low specificity, though controversial, could be that RDTs are more sensitive than microscopy, meaning that false positives were actually true positives [43]. Support for this conclusion may be found in Ratsimbasoa *et al*. who showed a higher specificity if RDTs were compared with PCR than if RDTs were compared with microscopy [29].

The performance of RDTs did not seem to be much influenced by execution and interpretation of RDTs, as these showed generally good results. It should however be considered that all results were collected by (in)direct evaluation of the CHWs and this may have biased the outcome [45]. Job aid and training were factors positively associated with interpretation and execution of RDTs, however, this improvement could also be related to the selection bias in CHWs. That would imply CHWs perform better with higher educational background. Nevertheless progress may still be obtained by increased practice of RDTs during training [18,19], especially practice of interpretation of faint positives and invalid test results and practice of the steps that were found to be frequently problematic; these were collecting blood in the right way and in the right amount, dispensing buffer drops in the right amount and in the right well, registering patient data and waiting the correct time before reading test results [18-20]. It is unknown why these steps were more problematic. Only one study reported an event with high safety risk [18], but safety issues should always be well addressed during training. Furthermore, visual impairments may hamper the correct interpretation of RDTs [34] and therefore screening for visual impairments should be done before appointing new CHWs.

RDT performance could also have been influenced by 1) storage conditions [46], which stresses the need of a cold chain, or 2) type of RDT, because of variable intrinsic diagnostic qualities and differences in ease of execution and interpretation. Many types of RDTs are available and not enough evidence was found in this review to advise on a specific type of test. Guidance for RDT choice can be found in a published WHO report which shows an extended overview of all different malaria RDTs, although it should be noted that these were tested in regulated circumstances by laboratory technicians, thus not informing on end-user impact [47].

Adherence to test results and referral guidelines is important for safe and successful implementation of RDT-based CCMm and the reviewed literature highlighted that CHWs showed generally high levels of adherence to test results regarding treatment. This in contrast to professional healthcare workers, in whom adherence was found to be much more variable because they often rely on clinical judgment instead of RDT results [48-52] and the fear of false-negative RDT results may tempt them to treat negative patients, especially in the case of subjects with no or little malaria immunity [53]. It could be that these reasons also contributed to the few studies on CHWs who reported non-adherence to negative test results [20,27,30], especially because CHWs were previously instructed to regard any fever case as malaria. This is however unknown since CHW reasons for not adhering to negative test results were not investigated, although several studies stressed the importance of training and monitoring in order for CHWs to adhere to the study guidelines [17,18,23,24,26,28,30,32,33,40]. Interestingly, all studies with an iCCM design showed good adherence to negative RDT results. The provided alternatives for malaria treatment in iCCM, such as antibiotic treatment for pneumonia after diagnosis with a respiratory rate timer, could have contributed positively to this adherence.

Unfortunately, despite the importance of referring patients with danger signs, adherence to referral policy is scarcely addressed, with one study reporting low [20] and one reporting high adherence [25]. Again, why CHWs did not refer patients was not studied. From the patient perspective, two studies investigated referral completion and showed that many patients did not follow up on referral, although the big difference in magnitude between these studies is remarkable [33,39]. Possible barriers for patients to complete referral were found to be mostly due to transportation difficulties; a problem that is even bigger for health centre-based care and one that is not easily solved, although iCCM could reduce the overall need for referral. Other barriers were not thoroughly investigated; however an interesting finding was that RDT result apparently influenced referral completion [39].

Studies on morbidity and mortality lacked high quality evidence which impairs firm conclusions. CCMm should be beneficial compared with presumptive CCMm by decreasing overtreatment, and increasing diagnoses of other diseases. However, false negative RDT results could negate the beneficial effects. The low evidence is surprising, but may reflect the more challenging design needed, which should incorporate follow-up of all patients.

The effect on morbidity and mortality in the total population is dependent on acceptability and healthcare-seeking behaviour of the community, which was found to increase within the community after introduction of RDT-based CCMm. It should, however, be noted that the studies reporting on the uptake of CCMm with RDTs took place during a trial, which could influence the way people behave. This effect is better known as the Hawthorne effect [45]. Several aspects were mentioned as barriers of CCMm uptake in the reviewed studies. Even though CHWs are mostly selected by the community or community leaders and must often meet certain criteria of educational background, barriers related to distrust of the CHW are mentioned [37]. How often distrust is a problem and what this is based upon is unknown. Better understanding of distrust might help in correctly sensitizing the community to increase acceptance and uptake of CCMm. Other barriers are logistical factors such as distance and transport to CHW, unavailability of CHW and stock outs. As CCMm already brings malaria care closer to the patient’s home, problems of distance and transport are not specific for community based healthcare and are difficult to solve. Unavailability of the CHW might be related to too little remuneration, limiting CHWs in their time because they must take care of their livelihood. CHW remuneration is shortly discussed below. The issue of stock-outs seems to be a frequent problem for RDTs and anti-malarial drugs [18,20,33,38]. Next to the failure to provide patients the care they need at these moments, even the rumour of stock-out could prevent people from visiting CHWs [37]. The problem might be even bigger after trial termination because stock supply is usually more strictly regulated during study periods. Experience with stock management by CHWs may have a positive influence on the availability [20], but stock management by CHWs is not always the main barrier [33]. CHWs usually rely on nearby health facilities for their stock supply but, due to financial, logistical, political, and other factors, drug supply through the government health systems is often unreliable and shortages of essential drugs are common in health centres as well, especially in the most remote areas [54].

Another issue important for sustainability of RDT-based CCMm is CHWs’ motivation and remuneration. It is unfortunate that information is scarce and there is no evaluation of motivation and the remuneration alternatives on the subsequent retention of CHWs in RDT-based CCMm. However, respect by the community and spiritual outcomes seem to be important for motivation. This was also reported in a recent Cochrane review that informed on motivation for lay health workers involved in all types of healthcare interventions [55]. Altruism, social recognition, knowledge gain, and career development were mentioned as motivations. Discrepancies were found in opinions on payment as incentive; supporters thought their time investment and obtained skills should be compensated, opponents thought working in a profit-oriented way can evoke negative reactions within the community [56]. Non-monetary incentives, such as bicycles, uniforms, mobile phones, or health insurance were generally well appreciated [56,57].

The last important item for long-term implementation, in particular for policy makers, is cost-effectiveness. Cost-effectiveness is highly dependent on malaria transmission, infrastructure and existing health systems. It can be concluded from the reviewed literature that the possible monetary beneficial effects of RDT-based CCMm *versus* presumptive CCMm are the biggest in areas with low to moderate malaria prevalence, at least for the costs per correctly treated case. With declining malaria prevalence in many SSA countries, RDT-based CCMm will probably become increasingly cost-effective [58]. However, because of limitations in the study designs these cost-effectiveness analyses are not informative enough for policy makers. Most importantly not all health-related benefits were included, such as the effect on longer term morbidity and mortality. Also, the real-time information on malaria prevalence that can be obtained with RDT testing is of importance for malaria control programmes. Moreover, testing with RDTs enables CHWs to adequately treat other febrile diseases than malaria and is the first step in moving from CCMm to iCCM. Because of these additional benefits, cost-utility analyses on differences in disability or quality adjusted life years (DALYs or QALYs) would gain the most information on the actual effects for patients and the possible impact for society of RDT-based CCMm.

A number of limitations are present due to the scope of this review. First of all, for several outcomes only a few studies were found, limiting the amount of evidence. Moreover, the few studies found were sometimes of insufficient quality for the relevant outcome, such as for morbidity and mortality. In general, very few studies investigated the factors that could explain why some of the critical steps in CCMm are successful or not. Secondly, in contrast to outcomes of diagnostic quality, cost-effectiveness, or impact of intervention on morbidity or mortality, it was challenging to assess the risk of bias for the remaining outcomes, because criteria for quality assessment were not standardized. Thirdly, most outcomes were investigated in trial settings, in which logistics are frequently well arranged and motivation is high, but only until the end of the study period. The lack of implementation studies may therefore have caused a bias. Finally, the restriction of the review to CCMm prevented the use of studies on iCCM without distinguishable CCMm data, but this was the only way to establish the benefits and failures of the individual intervention for the fight against malaria. Nevertheless, integrated CCM is an important development that may lead to increased access to targeted healthcare, without jeopardizing but potentially even improving malaria care.

Although the data presented in this review give a wealth of information, several aspects warrant further studies. First of all, no firm conclusions can be made on the absolute impact of the test and treat policies on morbidity and mortality. Further studies appropriately designed to measure this effect are needed and should preferably include follow-up of all patients, including referred and RDT-negative patients to also estimate the change in detection of other febrile diseases. Proven effect on morbidity and mortality will help policy makers in their decision about implementation of the intervention.

Furthermore, this review has shown that social science contributions are scarce. This limits the understanding of the implementation of RDT-based CCMm in different local contexts. Multidisciplinary approaches in which biomedical and social sciences asses the intervention, both by qualitative and quantitative methods, are needed. Factors influencing adherence to test results, referral completion, acceptance and uptake of the intervention and CHW attrition need to be more thoroughly investigated. Moreover, research is required to assess the best and most sustainable way of (re)training and supervising CHWs.

Little is published on logistic and structural elements, which would allow the design of a sustainable programme. Therefore, the system of stock supply and stock management throughout the healthcare system should be outlined and analysed to detect the problem areas.

## Conclusions

Despite limitations of the currently available evidence, several recommendations can be made for the design and implementation of CCMm. It is most important that CHWs receive a training in which they have enough opportunity to practice the difficult steps and interpretation of RDTs to ensure adequate execution and interpretation of the tests and consequently ensure the most optimal test performance. Furthermore, the risks derived from potentially lower specificity may be outweighed if iCCM is implemented. A job aid, repeated training and supervision can subsequently enhance the overall performance of CHWs, including adherence to test results. Again, further improvement in adherence can be obtained by implementing iCCM. Community sensitization is needed to ensure comprehension of the intervention and trust in the skills of the CHW. Furthermore, it might stimulate adherence to treatment and referral advice. The stock management system needs to be elucidated and stock management training should be an integrated part in the CHW and health centre staff training.

Finally, since factors influencing cost-effectiveness are abundant and variable in different malaria-endemic areas and because the scarce number of studies available lack the inclusion of important benefits, an individual cost-effectiveness analysis is still needed for each area preparing for RDT-based CCMm implementation [58].

The implications raised in this review can be used to draft RDT-based CCMm or iCCM programmes and research projects, even for risk groups not explicitly addressed in most RDT-based CCMm studies, such as pregnant women. However, specific considerations would be in place depending on malaria pathogenesis, transmission dynamics, the existing healthcare structure and the local culture and social setting.

## Abbreviations

ACT: Artemisinin-combination therapy; CCMm: Community case management of malaria; CHW: Community health worker; CMD: Community medicine distributor; iCCM: Integrated community case management; RDT: Rapid diagnostic test; SSA: Sub-Saharan Africa; WHO: World Health Organization.

## Competing interests

The authors declare that they have no competing interests.

## Authors’ contributions

ER, PFM, HS, SD and KPG conceived the idea for this study. ER and PFM designed the study and performed the searches. ER and PFM performed the primary selection of eligible studies and subsequently the inclusion of studies and the critical appraisal. Data was collected and analysed by ER and PFM with support from SD and KPG for the social anthropological issues. Any disagreements in the aforementioned steps were resolved by discussion until agreement was reached. The first draft of this manuscript was written by ER. FP critically read and advised on the manuscript. All authors have contributed to the writing of the paper and all have reviewed and approved the final version.

## Supplementary Material

Additional file 1 **Search syntax.** Complete search syntax per database. Click here for file

Additional file 2 **Excluded full text articles.** List of excluded full text articles with reasons. Click here for file

Additional file 3 **Overview of included articles and reported outcomes.** All included studies are listed with indication of the critical steps they report on. Click here for file

Additional file 4 **Eight critical steps in test execution.** Description of the eight critical steps that were used for judging test execution in the study of Counihan *et al.*[18]. Click here for file
