# Supplementary material for: Success or failure of critical steps in community case management of malaria with rapid diagnostic tests: a systematic review
Source: Malar J. 2014 Jun 12;13:229. doi: 10.1186/1475-2875-13-229 (PMC4084582; doi:10.1186/1475-2875-13-229)
Supplement: Additional file 1 — Search syntax. Complete search syntax per database. [file 1475-2875-13-229-S1.pdf]

Additional file 1

'Search syntax'. Latest update 12th October 2013.

**Database**	**Search syntax**
**Medline**<br><br>**http://www.ncbi.nlm.nih.gov/pubmed/**	malaria[mesh] OR malaria[tiab] OR plasmodium falciparum[tiab] OR p falciparum[tiab]<br><br>**AND**<br><br>mass screening[mesh] OR screen*[tiab] OR sst[tiab] OR rdt[tiab] OR rapid diagnos*[tiab] OR (rapid[tiab] AND test*[tiab]) OR (rapid antigen test*[tiab])<br><br>**AND**<br><br>home based[tiab] OR home management[tiab] OR community case management[tiab] OR community management[tiab] OR community based[tiab] OR hmm[tiab] OR community health[tiab] OR home health worker*[tiab] OR health community worker*[tiab] OR home case management[tiab] OR community directed[tiab] OR village health worker*[tiab] OR Community Malaria Volunteer*[tiab] OR community owned resource person*[tiab] OR village malaria worker*[tiab] OR health extension worker*[tiab] OR malaria control assistant*[tiab] OR community malaria volunteer*[tiab] OR "Community Health Workers"[Mesh] OR "Home Health Aides"[Mesh]
**Embase**	'malaria'/exp OR 'plasmodium falciparum'/exp OR malaria:ab,ti OR (plasmodium:ab,ti AND falciparum:ab,ti) OR (p:ab,ti AND falciparum:ab,ti) AND [embase]/lim<br><br>**AND**<br><br>'screening'/exp OR screen*:ab,ti OR sst:ab,ti OR rdt:ab,ti OR (rapid NEXT/1 diagnos*):ab,ti OR (rapid NEXT/4 test*):ab,ti OR ('rapid antigen' NEXT/1 test*):ab,ti AND [embase]/lim<br><br>**AND**

	'health auxiliary'/exp OR (home NEXT/1 based):ab,ti OR (home NEXT/1 management):ab,ti OR ('community case' NEXT/1 management):ab,ti OR (community NEXT/1 management):ab,ti OR (community NEXT/1 based):ab,ti OR hmm:ab,ti OR (community NEXT/1 health):ab,ti OR ('home health' NEXT/1 worker*):ab,ti OR ('health community' NEXT/1 worker*):ab,ti OR ('home case' NEXT/1 management):ab,ti OR (community NEXT/1 directed):ab,ti OR ('village health' NEXT/1 worker*):ab,ti OR ('community malaria' NEXT/1 volunteer*):ab,ti OR ('community owned resource' NEXT/1 person*):ab,ti OR ('village malaria' NEXT/1 worker*):ab,ti OR ('health extension' NEXT/1 worker*):ab,ti OR ('malaria control' NEXT/1 assistant*):ab,ti OR ('malaria village' NEXT/1 worker*):ab,ti AND [embase]/lim
**Cochrane**<br><br>**http://www.thecochranelibrary.com**	malaria or "plasmodium falciparum" or "p falciparum":ti,ab,kw in Cochrane Reviews (Reviews and Protocols) and Trials (Word variations have been searched) OR [Malaria] explode all trees OR [Malaria, Falciparum] explode all trees OR [Plasmodium falciparum] explode all trees<br>**AND**<br>screen* or sst or rdt or (rapid next diagnos*) or (rapid next test*) or ("rapid antigen" next test*):ti,ab,kw (Word variations have been searched) OR [Mass Screening] explode all trees<br>**AND**<br>"home based" or "home management" or "community case management" or "community management" or "community based" or hmm or "community health" or ("home health" next worker*) or ("health community" next

	worker*) or "home case management" or "community directed" or (" village health" next worker*) or ("Community Malaria" next Volunteer*) or ("Community owned resource" next person*) or ("village malaria" next worker*) or ("health extension" next worker*) or ("malaria control" next assistant*):ti,ab,kw (Word variations have been searched) OR [Community Health Workers] explode all trees
**MIP library**<br><br>**http://library.mip-consortium.org/**	malaria:TI or malaria:AB or (plasmodium falciparum:TI) or (plasmodium falciparum:AB) or (p falciparum:TI) or (p falciparum:AB)<br>**AND**<br>screen*:TI or screen*:AB or sst:TI or sst:AB or rdt:TI or rdt:AB or ((rapid:TI) next diagnos*:TI) or ((rapid:AB) next diagnos*:AB) or ((rapid:TI) next test*:TI) or ((rapid:AB) next test*:AB) or ((rapid antigen:TI) next test*:TI) or ((rapid antigen:AB) next test*:AB)<br>**AND**<br>(home based:TI) or (home based:AB) or (home management:TI) or (home management:AB) or (community casemanagement:TI) or (community case management:AB) or (community management:TI) or (community management:AB) or (community based:TI) or (community based:AB) or hmm:TI or hmm:AB or (community health:TI) or (community health:AB) or ((home health:TI) next worker*:TI) or ((home healh:AB) next worker*:AB) or ((health community:TI) next worker*:TI) or ((health community:AB) next worker*:AB) or (home case management:TI) or (home case management:AB) or (community directed:TI) or (community directed:AB) or ((village health:TI) next worker*:TI) or ((village health:AB)

	next worker*:AB) or ((community malaria:TI) next volunteer:TI) or ((community malaria:AB) next volunteer:AB) or ((community owned resource:TI) next person*:TI) or ((community owned resource:AB) next person*:AB) or ((village malaria:TI) worker*:TI) or ((village malaria:AB) worker*:AB) or ((health extension:TI) next worker*:TI) or ((health extension:AB) next worker*:AB) or ((malaria control:TI) next assistant*:TI) or ((malaria control:AB) next assistant*:AB) or ((malaria village:TI) next worker*:TI) or ((malaria village:AB) next worker*:AB)
