# Supplementary material for: Success or failure of critical steps in community case management of malaria with rapid diagnostic tests: a systematic review
Source: Malar J. 2014 Jun 12;13:229. doi: 10.1186/1475-2875-13-229 (PMC4084582; doi:10.1186/1475-2875-13-229)
Supplement: Additional file 2 — Excluded full text articles. List of excluded full text articles with reasons. [file 1475-2875-13-229-S2.pdf]

**Additional file 2**

'Excluded articles with reasons'.

| **Excluded article** | **Reason for exclusion** |
|---|---|
| Alfred Tiono 2010 et al. | Abstract. Author referred to Tiono *et al.* 2013 |
| Ashton et al. | Only opinion of CHWs on ease of handling of RDT. |
| Betson 2011 et al. | Abstract. |
| Bjorkman 2011 et al. | Abstract. Study described in Msellem *et al.* 2009 (PLoS Med): no use of CHWs. |
| Blanas et al. 2010 | Abstract. Full study described in Blanas *et al.* 2013 |
| Brieger et al. 2011 | Abstract. |
| Buwembo 2012 et al. | Abstract. |
| De Smet et al. | Abstract. Author suggested Tayler-Smith *et al.* 2011. |
| Diarra 2012 et al. | Community clinic health workers. No response on question whether these met the definition of CHWs. |
| Diouf 2010 et al. | Abstract. Not specific on RDT based home-management intervention. |
| Gerstl 2010 et al. | No CHWs. |
| Gerstl 2009 et al. | Abstract. Full text in Malaria J 2010: not about CHWs. |
| Hopkins 2011 et al. | Only data available for all health workers combined, not separately for CHWs. |
| Iyer 2011 et al. | Abstract. About integrated CCM. |
| Jean et al. 2011 | Abstract, full study described in Tine *et al.* 2011 (Malaria J) |
| Landoh 2012 et al. | Morbidity study not focused on RDT based CCMm |
| Luchavez et al. 2007 | Not situated in SSA and not the right outcome. |
| Masanja et al. 2010 | Only data available for all health workers combined, not separately for 'volunteers'. |
| Muhindo et al. 2012 | No CHWs performing RDTs. |
| Ndiaye et al. 2011 | Abstract. Described in Ndiaye *et al*. 2013 |
| Ndiaye et al. 2012 | Abstract. Described in Ndiaye *et al*. 2013 |
| Ndyomugyenyi et al. 2012 | Abstract. |
| Ngasala 2011 et al. | RDTs only used for first screening, treatment and follow-up based on subsequent microscopy result so no relevant outcomes. |
| Orji et al. 2012 | Abstract. |
| Ouatarra et al. 2011 | No CHWs. |
| Pagnoni et al. 2011 | Abstract, full study described in Mukanga *et al.* 2012 (multi-country study) |
| Ponsar et al. 2009 | Abstract. |
| Roger et al. 2011 | Abstract, full study by Tine *et al.* 2011 |
| Rutta et al. 2009 | Abstract, full study by Rutta *et al.* 2012. |
| Shekalaghe et al. 2013 | No CHWs. |
| Silumbe et al. 2012 | Abstract. |
| Thiam et al. 2012 (AMJTMH) | Abstract. |
| Thiam et al. 2012 (AJTMH) | Abstract. Full study in Thiam *et al.* 2012 (Malaria J) |
| Thiam et al. 2011 | Abstract. Full study in Thiam *et al.* 2012 (Malaria J) |
| Tine 2011 et al. | Intervention directed at IPT instead of RDT based CCMm. |
| Willcox et al. | RDTs not performed by CHWs. |
| Yeboah-Antwi et al. 2009 | Abstract. Full study described in Yeboah-Antwi *et al.* 2010. |
