# Supplementary material for: Success or failure of critical steps in community case management of malaria with rapid diagnostic tests: a systematic review
Source: Malar J. 2014 Jun 12;13:229. doi: 10.1186/1475-2875-13-229 (PMC4084582; doi:10.1186/1475-2875-13-229)
Supplement: Additional file 3 — Overview of included articles and reported outcomes. All included studies are listed with indication of the critical steps they report on. [file 1475-2875-13-229-S3.pdf]

Additional file 3

‘Overview of included studies and reported outcomes.’

Study	Country	Participants	Study design	RDT performance	RDT interpretation	RDT execution	Adherence to test results by CHWs	Morbidity / mortality	Acceptability by community	Uptake by community	Stock outs	CHW incentives	Cost – (effectiveness)
Blanas [34] 2013	Senegal	All care-seeking patients	Pre- and post-training assessments of CHWs. Post-training assessment two months after training.						x		x		
Chanda [20] 2011(1)$_a$	Zambia	All care-seeking patients	One-year evaluation on the costs and effects of RDT-based CCMm *versus* health centre-based care.				x						x
Chanda [35] 2011(2)$_a$	Zambia	All care-seeking patients	Prospective 1.5-year evaluation of CHWs competence in RDT-based CCMm				x		x		x		
Chinkhumba [27] 2010	Malawi	Patients ≥5 years with (history of) fever	Cross-sectional study on RDT use by CHWs	x			x						
Counihan [28] 2012	Zambia	All care-seeking patients	Prospective 1-year evaluation on RDT use by CHWs		x	x					x		
Elmardi [24] 2009	Sudan	All care-seeking patients	Pre- and post-intervention assessment (duration 9 months) on RDT-based CCMm.				x	x	x	x	x	x	
Hamer [16] 2012$_b$ &	Zambia	Patients 6 months-5 years with fever or	Cluster randomized controlled trial. Intervention CHWs performed RDT-based CCMm and				x			x		x	

Yeboah-Antwi [15] 2010$_b$		respiratory symptoms.	pneumonia treatment with amoxicillin. Control CHWs treated malaria presumptively and referred pneumonia patients.										
Harvey [30] 2008	Zambia	Febrile patients.	Study on competence of CHWs in RDT performance directly after 3 hours' training.		x	x							
Hawkes [18] 2009	DRC	Patients 0-14 years with fever	One-week evaluation on RDT-based CCMm.		x	x							x
Ishengoma [25] 2011$_c$	Tanzania	Patients with (history of) fever	Longitudinal study (5 years) on RDT-based CCMm for patients ≥5 years and presumptive treatment for patients <5 years	x			x						
Lemma [37] 2010	Ethiopia	Care-seeking patients ≥1 year	Two-year study. Year 1 all intervention CHWs presumptively treated malaria. Control areas had no CCMm. Year 2 half of intervention CHWs performed RDT-based CCMm.							x			
Lemma [19] 2011	Ethiopia	Patients with (history of) fever	Evaluation of three CCMm approaches. 1) Diagnosis of species-specific malaria + treatment; 2) Diagnosis of only *P. falciparum* + treatment; 3) Presumptive diagnosis + treatment	x									x
Mubi [21] 2011	Tanzania	Patients ≥3 months with (history of) fever	Randomized crossover trial. One group of CHWs performed RDT-based CCMm, other group treated malaria presumptively. Groups alternated every week.	x	x		x	x	x				
Mukanga [31] 2010	Uganda	Caregivers of children <5 years.	Survey among community members prior to implementation of RDT-based CCMm.						x	x			
Mukanga [29] 2011$_d$	Uganda	Patients <5 years with (history of) fever	Study on competence of CHWs to use RDTs. Assessment 3 days-2 weeks after training. 13 consultations observed.		x	x	x						
Mukanga [33] 2012$_d$	Uganda	Caregivers of patients <5 years	Cross-sectional study on caregivers' opinion on intervention CHWs performing RDT-based CCMm and treating pneumonia with amoxicillin.						x	x			

Mukanga [57] 2012$_d$	Burkina Faso, Ghana, Uganda	Patients 4-59 months with (history of) fever	Cluster randomized controlled trial. Intervention CHWs performed RDT-based CCMm + pneumonia diagnosis (respiratory rate) and treatment. Control CHWs treated malaria presumptively, in Ghana they also treated pneumonia on clinical diagnosis.				x						
Ndiaye [17] 2013	Senegal	All care-seeking patients	Evaluation of two types of CHWs on performance of RDT-based CCMm.			x	x				x		
Nsagha [32] 2012	Cameroon	People from different sociodemographic backgrounds	Focus group discussions and in-depth interviews on RDT-based CCMm before implementation.						x				
Premji [58] 1994	Tanzania	Patients <42 months who are regularly tested for malaria.	Study on RDT performance in hands of CHWs.	x									
Ratsimbasoa [26] 2012	Madagascar	Patients 2-59 months with (history of) fever	Evaluation of one year intervention of RDT-based CCMm.	x									
Rutta [23] 2012$_c$	Tanzania	All care-seeking patients.	Pre- and post-intervention survey (study of 4 years) on RDT-based CCMm for patients ≥5 years and presumptive treatment for patients <5 years.					x					
Tayler-Smith [14] 2011	Chad	Chad: patients <15 years	Evaluation of up to 5 years of RDT-based CCMm.							x			
Tiono [13] 2013$_d$	Burkina Faso	Children aged 6-59 months with (history of) fever	Study on RDT performance if used by CHWs from the intervention arm of a cluster-RCT	x									
Thiam [22] 2012	Senegal	Patients with suspected malaria.	Non-randomized controlled trial. 25 rural districts with RDT-based CCMm as intervention, control areas with no CCMm at all.				x	x					
Thomson [36]	Sierra Leone	Patients 3-59	Survey on referral based on record reviews over						x				

2011		months and pregnant women in 2nd and 3rd trimester.	three months in an area with recently introduced RDT-based CCMm.										
Total				7	5	5	12	4	8	6	5	2	4

a-d Articles with the same letter are based on the same study.
