# Supplementary material for: Success or failure of critical steps in community case management of malaria with rapid diagnostic tests: a systematic review
Source: Malar J. 2014 Jun 12;13:229. doi: 10.1186/1475-2875-13-229 (PMC4084582; doi:10.1186/1475-2875-13-229)
Supplement: Additional file 4 — Eight critical steps in test execution. Description of the eight critical steps that were used for judging test execution in the study of Counihan et al.[18]. [file 1475-2875-13-229-S4.pdf]

Additional file 4

Counihan *et al.*: **Community health workers use malaria rapid diagnostic tests (RDTs) safely and accurately: results of a longitudinal study in Zambia.** *Am J Trop Med Hyg* 2012, **87**:57–63

19 steps required to correctly and safely prepare a rapid diagnostic test (RDT) (critical steps noted in boldface type).

1. Assemble new test packet, swab, buffer, pipette, lancet and gloves.
2. **Put on new pair of gloves.**
3. Check expiry date on package.
4. Check desiccant sachet is still dry (do not include answer in total score).
5. Write patient's name on cassette.
6. Place cassette on a level surface.
7. **Clean finger with antiseptic/alcohol.**
8. Allow finger to dry before pricking it.
9. **Use a sterile lancet for finger prick.**
10. Puncture the side of the ball of the finger
11. **Dispose of lancet in sharps bin immediately after pricking finger.**
12. Collect blood with the enclosed pipette making sure to fill close to the first cross line.
13. **Using the pipette, blot blood onto the pad in the smaller well.**
14. Dispose of pipette in sharps container immediately.
15. **Dispense 5 drops of clearing buffer into the larger well.**
16. **Wait 15 minutes before reading *negative* results.***
17. **Read test results correctly.**
18. Record results in CHW register.
19. Dispose of non-sharps (gloves, wrappers, alcohol swab, and desiccant) safely.

*Positive results may be read before 15 minutes if control line has also appeared. Results should not be read after 30 minutes.
